# Significant Effect of Raloxifene on Bone in Hemodialysis Patients

**DOI:** 10.5812/ijem.6251

**Published:** 2012-09-30

**Authors:** Pernille Ravn

**Affiliations:** 1 Department of Gynecology and Obstetrics, Gynecologic Endocrinology Unit, Odense University Hospital, Odense C, Denmark

**Keywords:** Raloxifene, Hemodialysis Patients

Dear Editor,

Attention should be drawn to the study by colleagues Saito O et al. published in the International journal of Endocrinology and Metabolism (1). The study suggests that raloxifene has a significant beneficial effect on bone in postmenopausal women with type 2 diabetes (2DM) undergoing hemodialysis (1). Hemodialysis patients are known to have a high risk of low bone mineral density and bone fractures (2). Furthermore, patients with 2DM are at high risk of bone fracture even if their BMD is normal or high, a phenomenon which might be explained by poor bone quality (3). Patients with 2DM and a need for hemodialysis at thus at specific risk, and it is thus highly relevant to search for new treatments that protect bone mass in this group of patients. Raloxifene is a selective estrogen receptor modulator (SERM) used in the prevention and treatment of postmenopausal osteoporosis (4). The present study by Saito O et al. is the first report of raloxifene for hemodialysis patients with 2DM using the same regimen as recommended for prevention and treatment of postmenopausal osteoporosis (3). The 1-year study included 60 postmenopausal women, 30 women with 2DM and 30 women without 2DM. The mean age was 66 years and the mean time since the start of hemodialysis was 5 years. The women were randomized to raloxifene 60 mg per day or no treatment securing a comparable number of patients in each of the 4 groups: plus minus raloxifene treatment, plus minus 2DM. Irrespective of whether the women had 2DM or not, the marker of bone resorption, N-terminal cross-linking telopeptide of type I collagen, NTx, a widely accepted marker of bone resorption (5), decreased by 13-20% whereas NTX increased by 2-3 % in the control groups receiving no raloxifene treatment. This corresponded to an increase of 0.5-1 % in heel speed of sound (SOS), a marker correlated with bone mineral density (BMD) (6), whereas SOS decreased by 0.8 % in the control group. The study thus indicates a uniform suppression of the increased bone resorption observed in hemodialysis patients (2). Although the study was only one year of duration the results are significant. The incidence of side effects was low, an aspect which is specifically important to this vulnerable group of patients. The effect of raloxifene was comparable to the effect seen in otherwise healthy postmenopausal women (3) suggesting a beneficial effect of raloxifene on bone regardless of the presence of 2DM or need for hemodialysis. Other studies have shown that a reduction in bone resorption and an increase in BMD are associated with a protection against fractures (5, 6). As the fracture rate was not obtained in this short-term study, this aspect, however, remains to be investigated in longer-term studies.
